# Draft genome sequence of *Priestia megaterium* strain HS-1 isolated from sea cucumber (*Apostichopus japonicus*) intestine

**DOI:** 10.1128/mra.01014-25

**Published:** 2025-11-24

**Authors:** Qingli Zhang, Lihua Zhao, Xiaoshan Wang, Tingting Gao, Tingting Tang, Mingcong Huang, Tingting Song, Zichu Zhao

**Affiliations:** 1 Department of Medicine, Qingdao Binhai University 563650, Qingdao, Shandong, China; 2 College of Agriculture and Animal Husbandry, Qinghai University 207475, Xining, Qinghai, China; Montana State University, Bozeman, Montana, USA

**Keywords:** *Priestia megaterium*, sea cucumber, genome

## Abstract

The draft genome of *Priestia megaterium* strain HS-1, isolated from sea cucumber intestine, is 5.2 Mb with 38% G+C, 30 contigs, 5,375 genes, 5,266 CDSs (5,205 protein-coding), 23 rRNAs, 78 tRNAs, and 8 ncRNAs.

## ANNOUNCEMENT

Sea cucumbers, as filter feeders, harbor intestines enriched with benthic microbes, forming a unique microbial community. This specialized microenvironment, characterized by high salinity and low oxygen levels, shapes the composition and function of their symbionts. Bacillus species dominate the isolated microbes from samples such as the body wall and intestines (1). *Priestia megaterium,* formerly known as *Bacillus megaterium*, is a Gram-positive aerobic rod that has been reclassified (2, 3). Here, *Priestia megaterium* strain HS-1, isolated from the intestines of sea cucumbers (samples: Yellow Sea, Qingdao, 35°46′14.398″ N, 120°09′41.267″ E), offers a model for host–microbe interplay and marine microbial mining.

Sea cucumbers (collected on 16 April 2025) were surface-cleaned with sterile saline. Intact intestines were aseptically excised, transferred to sterile Petri dishes, and rinsed two to three times. Each intestine was then longitudinally incised, rinsed gently to remove luminal contents, minced, and homogenized using a sterile grinder. Ten grams of the minced sea cucumber intestine were homogenized in 90 mL of sterile saline, followed by vortexing for 5 min to prepare a 10⁻¹ stock suspension. Serial dilutions (10⁻²–10⁻⁶) were subsequently prepared, and 0.1 mL of each dilution was spread in triplicate onto LB agar plates (Hopebio, Qingdao, China; composition: 10 g tryptone, 5 g yeast extract, and 10 g NaCl per liter). The inoculated plates were aerobically incubated at 37°C for 24 h. Isolated colonies were subjected to Gram staining and endospore screening. Endospore-forming rod-shaped bacteria were purified via triple streaking on LB agar, then cultured in LB broth (37°C, 100 rpm, 24 h) and identified by 16S rRNA gene sequencing (Table 1).

**TABLE 1:** 16S rRNA gene and genome assembly statistics of *Priestia megaterium* strain HS-1

Category	Specific content
16S rRNA gene sequencing	
GenBank accession	PX213457.1
Forward primer F(27) (5′–3′)	AGAGTTTGATCCTGGCTCAG
Reverse primer R(1492) (5′–3′)	CGGCTACCTTGTTACGACTT
Reaction mixture (25 µL)	1 µL DNA template (10-100 ng), 0.8 µL each primer (10 µmol/L), 12.5 µL 2 × PrimeSTAR HS DNA Polymerasex (Cat. R010A, Takara, China) and 9.9 µL ddH_2_O
Amplification condition	95°C 5 min and 32 cycles of 95°C 30 s, 55°C 45 s, and 72°C 90 s , 72°C 10 min
Sequencing kit and system	BigDye Terminator v1.1 (4336701, ThermoFisher) and ABI3730XL (Applied Biosystems, USA)
Genome assembly feature	
GenBank assembly accession	JBPZPF000000000
Total length (bp)	5,192,250
No. of contigs	30
Scaffold *N*_50_	945,234 bp
GC content (%)	38
Genome coverage	386.0x
Total genes	5,375
CDSs (total)	5,266
CDSs (with protein)	5,205
rRNAs (5S, 16S, 23S)	8, 10, 5
Complete rRNAs (5S)	3
Partial rRNAs (5S, 16S, 23S)	5, 10, 5
tRNAs	78
ncRNAs	8
Pseudogenes	61

Genomic DNA was extracted with the MagPure Bacterial DNA Kit (D6361-02; Magen, China). DNA was sheared to 200–400 bp, end-repaired, 3′-adenylated, adapter-ligated, PCR-amplified, and size-selected with Hieff NGS DNA Selection Beads (Cat# 12601) according to the manufacturer’s instructions. Libraries were quantified by Qubit v.4.0 fluorometry (Q33226; Thermo; default parameters were used except where otherwise noted) using the high-sensitivity kit, size-verified on a 2% agarose gel, and paired-end sequenced (2 × 150 bp) on an Illumina NovaSeq 6000 (Sangon Biotech, China) (4), yielding 2.14 billion raw paired-end reads. Raw reads were trimmed with fastp 0.23.0 (parameters: -q 15u 40n 5 -e 0L 35w 3) (5) and *de novo* assembled with SPAdes v.3.5.0 (parameters: --careful --k 33,55,77,99) (6). Sequences were polished with PrInSeS-G v.1.0.0 (7), and repeats were annotated using RepeatModeler v.2.0.6 and RepeatMasker v.4.1.5 (8, 9). Genome annotation (CDSs, tRNAs, rRNAs, ncRNAs, and pseudogenes) was performed with NCBI PGAP v.6.10 and Prokka v.1.10 (10, 11). Functional classification of predicted proteins employed COG (12) and KEGG (13).

The draft genome of *Priestia megaterium* strain HS-1 comprises 5.2 Mb assembled into 30 contigs (*N*_50_ = 945.2 kb, G + C = 38%). Annotation revealed 5,375 genes, 5,266 CDSs, of which 5,205 are protein-coding, along with 23 rRNAs, 78 tRNAs, and 8 ncRNAs. Mean sequencing depth was 386.0×. CheckM v.1.2.4 estimated gencompleteness at 94.49% with 3.68% contamination (14).

## Data Availability

The genome sequence for *Priestia megaterium* strain HS-1 has been deposited in GenBank under the accession number JBPZPF000000000, BioProject number PRJNA1300951, BioSample number SAMN50431476, and SRA number SRR34858341.

## References

[1] Chen L, Wang XY, Liu RZ, Wang GY. 2021. Culturable microorganisms associated with sea cucumbers and microbial natural products. Mar Drugs 19:461. doi:10.3390/md19080461 34436300 PMC8400260

[2] Andargie YE, Bashizi TF, Lim K, Getachew E, Shin J-H. 2025. Complete genome sequence of Priestia megaterium CIMT4 strain isolated from maize rhizosphere. Microbiol Resour Announc 14:e0112424. doi:10.1128/mra.01124-24 40539816 PMC12243543

[3] Choi S, Jung H, Kim Y, Han JA, Kim EY, Lee H-S. 2024. Draft genome sequence of Priestia megaterium strain IMGN3 derived from soil. Microbiol Resour Announc 13:e0045824. doi:10.1128/mra.00458-24 39162470 PMC11385723

[4] Minoche AE, Dohm JC, Himmelbauer H. 2011. Evaluation of genomic high-throughput sequencing data generated on illumina HiSeq and genome analyzer systems. Genome Biol 12:R112. doi:10.1186/gb-2011-12-11-r112 22067484 PMC3334598

[5] Chen S, Zhou Y, Chen Y, Gu J. 2018. Fastp: an ultra-fast all-in-one FASTQ preprocessor. Bioinformatics 34:i884–i890. doi:10.1101/274100 30423086 PMC6129281

[6] Bankevich A, Nurk S, Antipov D, Gurevich AA, Dvorkin M, Kulikov AS, Lesin VM, Nikolenko SI, Pham S, Prjibelski AD, Pyshkin AV, Sirotkin AV, Vyahhi N, Tesler G, Alekseyev MA, Pevzner PA. 2012. SPAdes: a new genome assembly algorithm and its applications to single-cell sequencing. J Comput Biol 19:455–477. doi:10.1089/cmb.2012.0021 22506599 PMC3342519

[7] Massouras A, Hens K, Gubelmann C, Uplekar S, Decouttere F, Rougemont J, Cole ST, Deplancke B. 2010. Primer-initiated sequence synthesis to detect and assemble structural variants. Nat Methods 7:485–486. doi:10.1038/nmeth.f.308 20543844

[8] Flynn JM, Hubley R, Goubert C, Rosen J, Clark AG, Feschotte C, Smit AF. 2020. RepeatModeler2 for automated genomic discovery of transposable element families. Proc Natl Acad Sci USA 117:9451–9457. doi:10.1073/pnas.1921046117 32300014 PMC7196820

[9] Tarailo-Graovac M, Chen N. 2009. Using RepeatMasker to identify repetitive elements in genomic sequences. Curr Protoc Bioinformatics Chapter 4:4. doi:10.1002/0471250953.bi0410s25 19274634

[10] Tatusova T, DiCuccio M, Badretdin A, Chetvernin V, Nawrocki EP, Zaslavsky L, Lomsadze A, Pruitt KD, Borodovsky M, Ostell J. 2016. NCBI prokaryotic genome annotation pipeline. Nucleic Acids Res 44:6614–6624. doi:10.1093/nar/gkw569 27342282 PMC5001611

[11] Seemann T. 2014. Prokka: rapid prokaryotic genome annotation. Bioinformatics 30:2068–2069. doi:10.1093/bioinformatics/btu153 24642063

[12] Tatusov RL, Galperin MY, Natale DA, Koonin EV. 2000. The COG database: a tool for genome-scale analysis of protein functions and evolution. Nucleic Acids Res 28:33–36. doi:10.1093/nar/28.1.33 10592175 PMC102395

[13] Kanehisa M, Goto S. 2000. KEGG: kyoto encyclopedia of genes and genomes. Nucleic Acids Res 28:27–30. doi:10.1093/nar/28.1.27 10592173 PMC102409

[14] Parks DH, Imelfort M, Skennerton CT, Hugenholtz P, Tyson GW. 2015. CheckM: assessing the quality of microbial genomes recovered from isolates, single cells, and metagenomes. Genome Res 25:1043–1055. doi:10.1101/gr.186072.114 25977477 PMC4484387

